# And … cut! Identifying chromatin features affecting CRISPR–Cas9 activity in plants

**DOI:** 10.1093/plphys/kiac348

**Published:** 2022-07-26

**Authors:** Marieke Dubois

**Affiliations:** Department of Plant Biotechnology and Bioinformatics, Ghent University, Ghent, Belgium; VIB Center for Plant Systems Biology, Ghent, Belgium

Since the end of the previous century, molecular plant biologists have tried to engineer the genome of model species and crops to better understand and improve diverse phenotypic traits. Genome engineering experienced a true revolution, roughly a decade ago, by the discovery of CRISPR–Cas9-mediated genome editing. In this technology, a bacterial DNA-cutting enzyme, the Cas9 nuclease, is directed to a specific position of the genome using a guide RNA (gRNA) with a sequence matching the target site (Jinek et al., 2012). Cas9 then introduces a double-stranded break in the target DNA, which can be repaired by nonhomologous end joining (NHEJ) or micro-homology end joining (MMEJ), two types of repair that often cause an insertion, deletion, or substitution of one or several nucleotides at the target site, possibly resulting in a loss- or gain-of-function mutation. CRISPR–Cas9 technology has the major advantage of generating mutations at a very specific, well-targeted site, compared to the classical approaches using random mutagenesis. Although the CRISPR technology has been successfully used for editing both crop genomes and model plants (Dima et al., 2022), editing efficiency can vary greatly (Kurokawa et al., 2021; Blomme et al., 2022). The underlying reasons of such variations in efficacy are not fully understood.

Multiple studies have been performed in various eukaryotic systems to uncover factors that can affect CRISPR–Cas9 efficacy. The target sequence was originally considered the key determinant for Cas9 activity. Nowadays, the genomic context of the target site and specific chromatin features around the target site are increasingly recognized as major determinants for Cas9 efficacy and for the outcome of the DNA repair, as demonstrated in mice (*Mus musculus*) and human (*Homo sapiens*) cell suspensions (Gisler et al., 2019; Schep et al., 2021). For example, repair by NHEJ predominantly occurs in open chromatin regions (euchromatin), while MMEJ is preferred in heterochromatin with specific histone methylation marks (H3K27me3) (Schep et al., 2021). Whether similar factors would affect gene editing efficacy and outcome in plants is an exciting question to be explored.

In the current issue of *Plant Physiology*, Weiss et al. (2022) took an unbiased, genome-wide approach to identify sequence-independent factors that affect CRISPR–Cas9 efficacy and outcome in whole Arabidopsis (*Arabidopsis thaliana*) plants. To eliminate the effect that the gRNA sequence would have on Cas9 efficacy, the authors aimed to target identical short sequences within their specific, natural genomic context. First, they established an algorithm to identify all possible CRISPR–Cas9 target sites in the Arabidopsis genome (Figure 1). These sites are determined by an NGG protospacer adjacent motif (PAM) recognition site at the 3′-end. Subsequently, they filtered the millions of identified sites to keep a handful of MultiCopy CRISPR sites, target sites that each occur at least 7 times in the genome, within diverse chromatin contexts. Finally, the authors targeted these MultiCopy sites with CRISPR–Cas9, followed by in-depth analysis of the outcome in stable Arabidopsis lines, in order to highlight nonsequence-related features determining the Cas9 activity (Figure 1).

**Figure 1:**
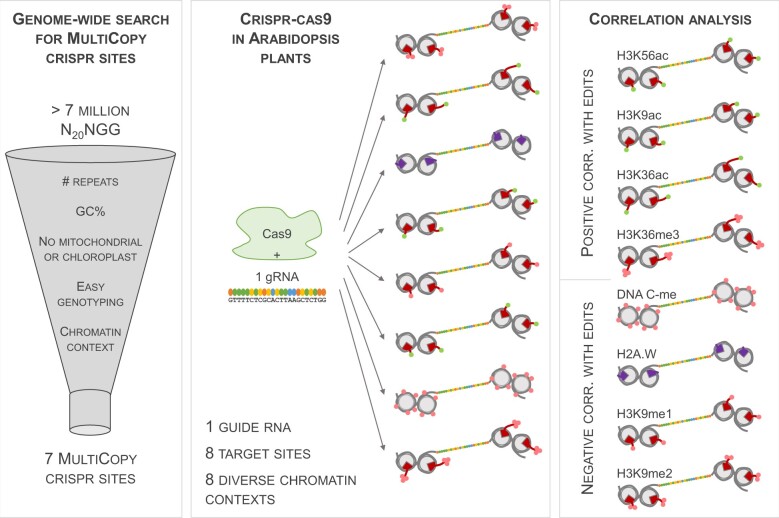
Strategy for identifying chromatin features associated with CRISPR–Cas9 efficacy in plants. In the work presented by Weiss et al. (2022), a genome-wide search of the Arabidopsis genome identified over 7 million putative CRISPR target sites (20 nt followed by NGG, the PAM recognition site). Subsequently, Weiss et al. filtered out sites with low number of repeats in the genome, with inappropriate GC content, and with target sequences in nonnuclear DNA. They further selected sites that could be genotyped with restriction enzymes and that occurred in diverse chromatin contexts. Doing so, seven candidate MultiCopy CRISPR sites were identified. Subsequently, a MultiCopy CRISPR site was targeted by a single gRNA, which guides the CRISPR–Cas9 machinery to the different genomic loci. These loci (eight depicted here) have diverse chromatin modifications and chromatin status (euchromatin or heterochromatin) but have an identical gRNA target sequence (multi-colored string). By quantifying the number of edits in stable Arabidopsis plants at the different loci, Weiss et al. identified sequence-independent features correlated with low or high CRISPR–Cas9 efficacy in plants. H2 = HISTONE2, H3 = HISTONE3, K = LYSINE, ac = Acetylation, C-me = CYTOSINE methylation, me1/2/3 = mono/di/tri-methylation.

For two of the tested MultiCopy CRISPR sites, the gRNA generated mutations at multiple sites in the Arabidopsis genome. Interestingly, while seven to eight sites were the potential targets of each of the two gRNAs, not all of them were edited with the same efficacy, and some sites were edited over 200 times more than others with the same sequence, demonstrating that the sequence itself is far from the only determinant of CRISPR–Cas9 efficacy in stable Arabidopsis plants. In addition, the sequence of the DNA flanking the target sites did not have a measurable impact on the CRISPR–Cas9 outcome. Instead, the accessibility of the chromatin, and the DNA and histone modifications that affect it, were most strongly correlated with the CRISPR–Cas9 outcome (Figure 1). Sites with low editing efficiency were associated with DNA hypermethylation or with occurrence of the histone variant H2A.W that promotes chromatin compaction in plants (Bourguet et al., 2021), and with methylation of H3K9, another typical heterochromatin feature. On the contrary, the highly edited sites were strongly correlated with open chromatin features, including HISTONE3 acetylation. However, two sites located in open chromatin were almost never edited, demonstrating that open chromatin alone is insufficient to ensure high CRISPR–Cas9 activity. More unexpectedly, besides controlling the chromatin structure and, thereby, the CRISPR–Cas9 efficacy, histone modifications also affected the outcome of DNA repair once Cas9 generated the DNA cut. By correlating the histone landscape around the gRNA sites with the occurrence of a substitution, deletion, or insertion upon repair, Weiss et al. observed that three HISTONE3-related features (H3K4me1, H3.1, and H3.3) are negatively correlated with insertional mutations at the edited site (Weiss et al., 2022).

Overall, the work of Weiss et al. (2022) emphasizes that nonsequence-related elements have a clear impact on the efficacy and outcome of CRISPR–Cas9-mediated mutagenesis in Arabidopsis. Findings in this study will help improve editing efficacy in plants. For example, lowly edited sites were associated with high DNA methylation and heterochromatin, and Weiss et al. (2022) demonstrated that reducing DNA methylation by multiple means can increase the Cas9-mediated mutagenesis frequency at lowly edited sites by at least two-fold. Whether these findings are applicable to other plants with higher economical value remains to be explored. Undoubtedly, the elegant and unbiased approach taken in this work will stimulate similar analyses in crops that are already routinely CRISPR edited to increase the editing efficacy or predict the editing outcome more precisely by considering the surrounding chromatin features.

## Funding

M.D. is a postdoctoral fellow of Flanders Research Foundation (FWO-12Q7919N).

*Conflict of interest statement*. None declared.
